# Features of increased malignancy in eosinophilic clear cell renal cell carcinoma

**DOI:** 10.1002/path.5532

**Published:** 2020-09-24

**Authors:** Helén Nilsson, David Lindgren, Håkan Axelson, Christian Brueffer, Lao H Saal, Jaana Lundgren, Martin E Johansson

**Affiliations:** ^1^ Department of Translational Medicine Lund University Malmö Sweden; ^2^ Division of Translational Cancer Research, Department of Laboratory Medicine Lund University Lund Sweden; ^3^ Division of Oncology, Department of Clinical Sciences Lund University Lund Sweden; ^4^ Clinical Pathology Sahlgrenska University Hospital Gothenburg Sweden; ^5^ Institute of Biomedicine, Department of Laboratory Medicine University of Gothenburg Gothenburg Sweden; ^6^ The Sahlgrenska Cancer Center University of Gothenburg Gothenburg Sweden

**Keywords:** renal cell carcinoma, clear cell, eosinophilic, kidney cancer, VHL, granular, mTOR, p53, mitochondria, electron microscopy, vasculature

## Abstract

Clear cell renal cell carcinoma (ccRCC) is the most common form of renal cancer. Due to inactivation of the von Hippel–Lindau tumour suppressor, the hypoxia‐inducible transcription factors (HIFs) are constitutively activated in these tumours, resulting in a pseudo‐hypoxic phenotype. The HIFs induce the expression of genes involved in angiogenesis and cell survival, but they also reset the cellular metabolism to protect cells from oxygen and nutrient deprivation. ccRCC tumours are highly vascularized and the cytoplasm of the cancer cells is filled with lipid droplets and glycogen, resulting in the histologically distinctive pale (clear) cytoplasm. Intratumoural heterogeneity may occur, and in some tumours, areas with granular, eosinophilic cytoplasm are found. Little is known regarding these traits and how they relate to the coexistent clear cell component, yet eosinophilic ccRCC is associated with higher grade and clinically more aggressive tumours. In this study, we have for the first time performed RNA sequencing comparing histologically verified clear cell and eosinophilic areas from ccRCC tissue, aiming to analyse the characteristics of these cell types. Findings from RNA sequencing were confirmed by immunohistochemical staining of biphasic ccRCC. We found that the eosinophilic phenotype displayed a higher proliferative drive and lower differentiation, and we confirmed a correlation to tumours of higher stage. We further identified mutations of the tumour suppressor p53 (*TP53*) exclusively in the eosinophilic ccRCC component, where mTORC1 activity was also elevated. Also, eosinophilic areas were less vascularized, yet harboured more abundant infiltrating immune cells. The cytoplasm of clear cell ccRCC cells was filled with lipids but had very low mitochondrial content, while the reverse was found in eosinophilic tissue. We herein suggest possible transcriptional mechanisms behind these phenomena. © 2020 The Authors. *The Journal of Pathology* published by John Wiley & Sons, Ltd. on behalf of The Pathological Society of Great Britain and Ireland.

## Introduction

Clear cell renal cell carcinoma (ccRCC) accounts for about 75% of all cases of kidney cancer in adults [1]. Surgery remains the only curative treatment. Targeted therapies may prolong survival but resistance often develops. ccRCC is characterized by histologically clear cells surrounded by dense arborizing vasculature. The apparently empty cytoplasm is an artefact; the initially massive amounts of cytoplasmic lipid and glycogen are washed out during histological processing, resulting in the defining clear cytoplasm [2]. Histopathological diagnosis, grading according to WHO/ISUP, and clinical staging remain prognostically most important [3]. The tumour suppressor protein von Hippel–Lindau (VHL) is inactivated in a majority of ccRCCs [4, 5]. VHL targets the hypoxia‐inducible factors (HIFs) for proteasomal degradation. The HIFs drive transcription of the cellular response to hypoxia, involving genes associated with angiogenesis, pH regulation, and cell survival [6]. In ccRCC, loss of VHL results in constitutive HIF activation, rendering ccRCC cells a pseudo‐hypoxic phenotype, resulting in the distinctive appearance.

As for many cancer forms, ccRCC displays intratumoural heterogeneity [7]. Tumour areas where cancer cells display eosinophilic cytoplasm can be found in parallel with clear cell areas. These tumours were previously sometimes referred to as granular RCC, offering differential diagnostic difficulties to other RCC forms with non‐clear cell histology. However, eosinophilic areas within ccRCCs share genetic features with the clear cell component, and today they are not considered as separate subtypes [3, 8]. It is known that eosinophilic areas often have higher nuclear grade and therefore may harbour worse prognosis [9]. Despite this, no studies have so far concurrently investigated the histological and possibly transcriptomic and prognostic differences of ccRCCs with concomitant clear cell and eosinophilic areas by direct comparison of the components. This study aims at a comprehensive characterization of a set of biphasic ccRCC cases and also compares these to ccRCCs without eosinophilic content.

## Materials and methods

### Tissue sampling

This study was performed in compliance with relevant ethical regulations and with permits from Lund University Ethics Committee (LU680‐08 and LU289‐07). Tissue was collected from nephrectomies performed at Malmö University Hospital, Sweden. Multiple tumour sampling was performed using 4‐mm biopsy punches. Each sample was divided in two. One piece was preserved in RNAlater (Ambion, Thermo Fisher Scientific, Waltham, MA, USA) for subsequent RNA extraction and the other was fixed in formalin for histological and immunohistochemical staining. Tumours were diagnosed as ccRCC by a pathologist specialized in urological pathology (MEJ), who also confirmed the histology of the cores as being of eosinophilic or clear cell phenotype based on haematoxylin and eosin (H&E) staining. Patient data are presented in supplementary material, Table S1. A validation set used for quantification of immunohistochemical staining was selected from a ccRCC tissue microarray. Cores were identified as eosinophilic or clear cell based on H&E staining. Double cores from ten cases of each phenotype were included in the validation cohort.

### RNA sequencing

Isolation and processing of RNA from tissue samples are described in supplementary material, Supplementary materials and methods. Analysis of the RNA sequencing data and additional data available from The Cancer Genome Atlas (TCGA) was performed as described previously [10] and in supplementary material, Supplementary materials and methods.

### Immunohistochemical and H&E staining

Formalin‐fixed, paraffin‐embedded tissue was cut into 4‐μm‐thick sections and stained with H&E according to standard procedures. A PT Link module (Dako GmbH, Jena, Germany) was used for deparaffinization and epitope retrieval. Immunohistochemical staining was performed using Autostainer Plus equipment (Dako). Antibodies are presented in supplementary material, Table S2. Staining was quantified using the Halo Image Analysis Platform (Indica Labs, Albuquerque, NM, USA).

### Mitochondrial DNA

Mitochondrial DNA content was determined as described previously [11]. In brief, total DNA was isolated using an Allprep RNA/DNA kit (Qiagen). Quantitative PCR was performed with primers for nuclear and mitochondrial encoded genes. Relative mitochondrial DNA content was determined by dividing the levels of mitochondrial DNA by the levels of selected nuclear encoded genes.

### Electron microscopy

Procedures for electron microscopy are described in supplementary material, Supplementary materials and methods.

## Results

### Eosinophilic ccRCC tissue

**Figure 1:**
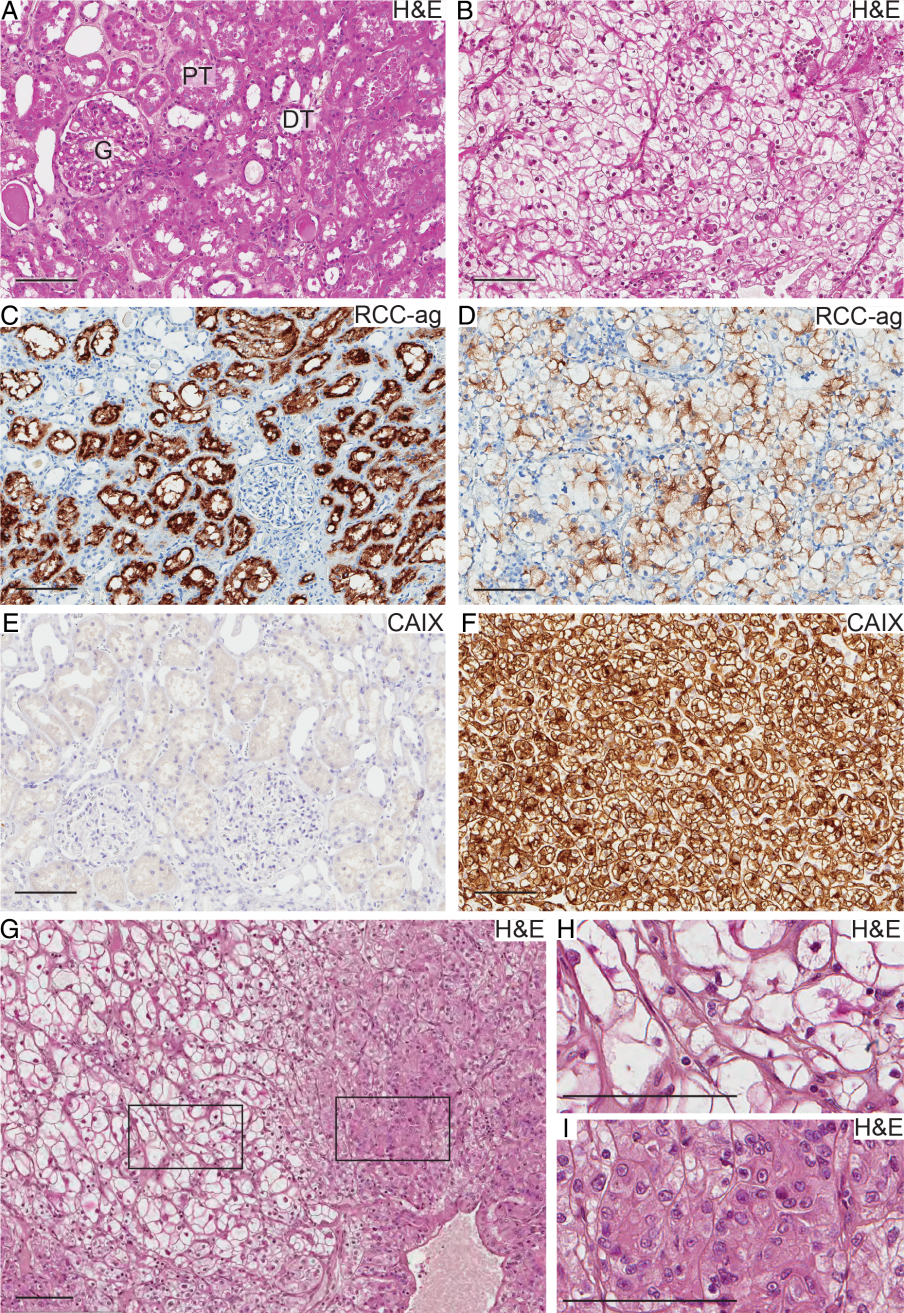
Histology of ccRCC tissue. (A–F) Normal kidney (A, C, E) and ccRCC tissue (B, D, F) from the same patient were stained with haematoxylin and eosin (A, B), for RCC antigen (C, D) and carbonic anhydrase 9 (CAIX) (E, F). (G) Overview of a biphasic ccRCC tissue sample. Heterogeneity with areas dominated by clear cells and eosinophilic cells, respectively, is visible. In H and I, the marked areas in G are enlarged. DT, distal tubule; G, glomerulus; PT, proximal tubule. Scale bars = 100 μm.

The histology of H&E‐stained normal kidney and ccRCC tissue from the same patient is presented in Figure 1A,B. The tumour displays the rich vasculature and clear cytoplasm of ccRCC. ccRCCs retain expression of the proximal tubular marker RCC‐antigen (Figure 1C,D), and the pseudo‐hypoxic phenotype results in upregulation of the HIF target carbonic anhydrase 9, not normally expressed in the kidney (Figure 1E,F). Biphasic ccRCC displaying areas of classical clear cells next to eosinophilic cells is shown in Figure 1G–I. The pink, granular cytoplasm of eosinophilic cells contrasts distinctly with the pale cytoplasm of clear cells.

Systematic evaluations of the frequency of ccRCCs with eosinophilic areas are few. Krishnan and Truong reported that tumour cells with granular cytoplasm were present in most, but predominant in 3 of 31 investigated tumours [12]. Among 146 ccRCCs resected at Malmö University Hospital between 2013 and 2017, eosinophilic cells were noted by the pathologist in 27% of cases. Scoring eosinophilic content in 98 ccRCC cases from The Cancer Genome Atlas (TCGA, https://www.cancer.gov) cohort indicated that in 21%, it was the predominant phenotype.

### The transcriptomic profile of eosinophilic ccRCC tissue

Histologically confirmed tissue from clear cell (ccRCC_Bcc) or eosinophilic (ccRCC_Beo) tumour areas from five biphasic ccRCCs was selected for RNA sequencing. The histology of these samples is presented in supplementary material, Figure S1. For comparison, ccRCCs without eosinophilic areas (ccRCC_cc) and normal kidney cortex (normal) were analysed. Tumour tissue was furthermore obtained from patients with VHL disease (ccRCC_VHL). These patients carry a monoallelic germline loss of VHL and are prone to develop ccRCC [13]. VHL disease patients are under regular surveillance to enable swift removal of appearing lesions. These tumours represent an early stage in ccRCC progression.

**Figure 2:**
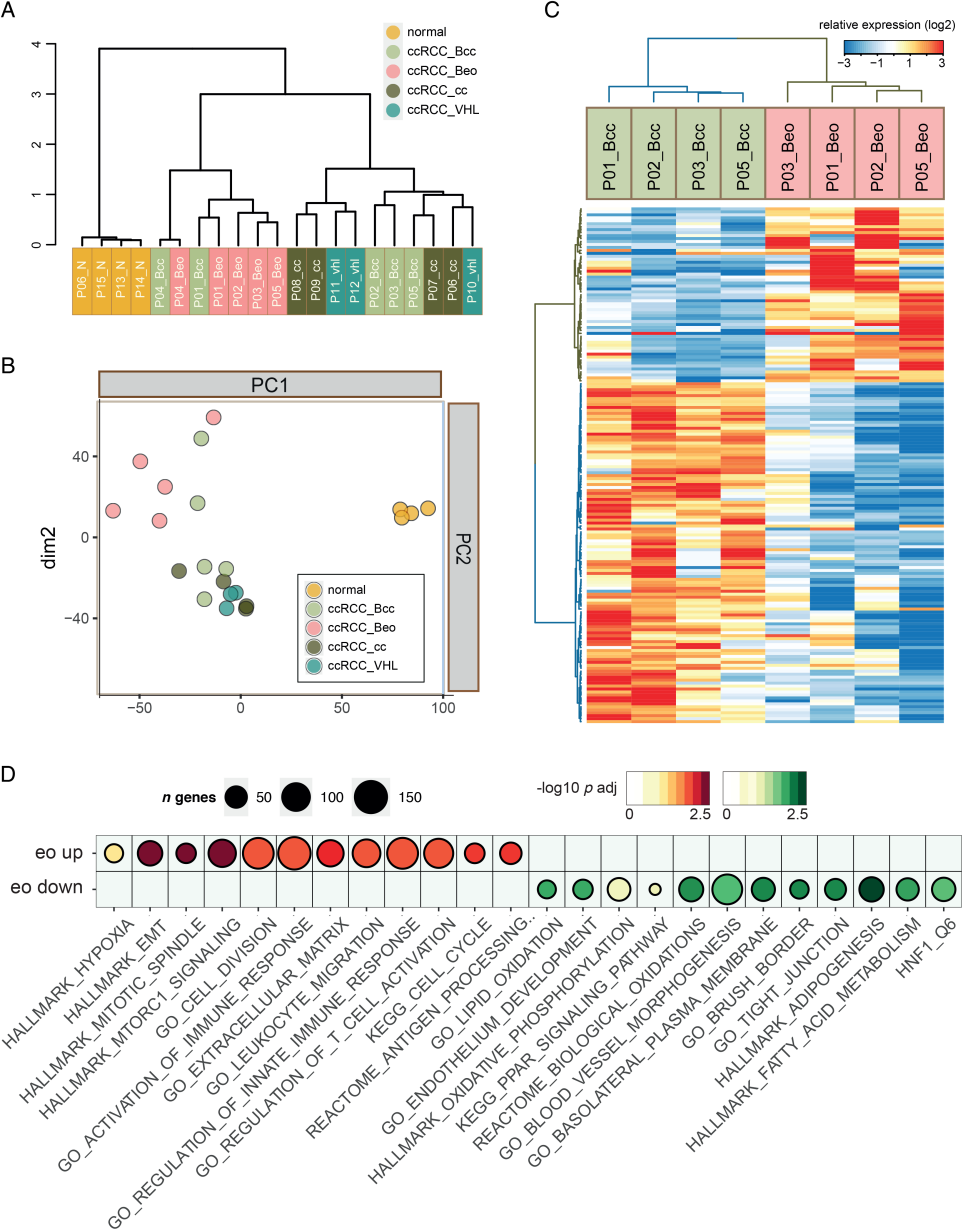
(A, B) Hierarchical cluster analysis (A) and principal component analysis (B) of sequenced samples. (C) Heatmap illustrating significantly differentially expressed genes in ccRCC_Beo compared with ccRCC_Bcc. (D) Selected Gene Ontology terms correlating to the eosinophilic (eo up) or the clear cell (eo down) phenotype are shown.

Hierarchical clustering and principal component analysis of sequenced samples are presented in Figure 2A,B. ccRCC_Bcc samples clustered closer together with ccRCC_cc and ccRCC_VHL, while ccRCC_Beo samples diverged. One exception was ccRCC_Bcc and ccRCC_Beo samples from patient 4 which clustered close together, separate from the other tumour samples (Figure 2A,B). Since these samples appeared more similar to each other than to the other samples of their respective phenotype, they were omitted from further analysis. As expected, normal kidney samples clustered together, well separated from all cancer samples.

Limma analysis identified 174 genes with phenotype‐specific expression when comparing ccRCC_Beo samples with ccRCC_Bcc (adjusted *p* < 0.05). Of these, 59 were significantly upregulated and 115 downregulated (Figure 2C and listed in supplementary material, Table S3). Several upregulated genes were associated with inflammation (for example, *IL6*, *CXCL5*, and *CCL20*) and cell cycle progression (*TPX2*, *PTTG1*). The gene cluster downregulated in ccRCC_Beo was dominated by membrane proteins and transporters associated with normal renal functions, such as *TMEM27*, a regulator of proximal tubule amino acid transport. Indeed, 14 of the significantly downregulated genes in eosinophilic components were members of the solute carrier gene family of membrane transport proteins, such as *SLC2A2*, *SLC3A1*, and *SLC17A1*. In addition, genes associated with lipid metabolism (*ACADL*, *ACSM2A*), mitochondria (*GLYAT*, *CYP1B1*), and tight junctions (*CLDN2*, *CRB3*) were found in the downregulated cluster.

**Figure 3:**
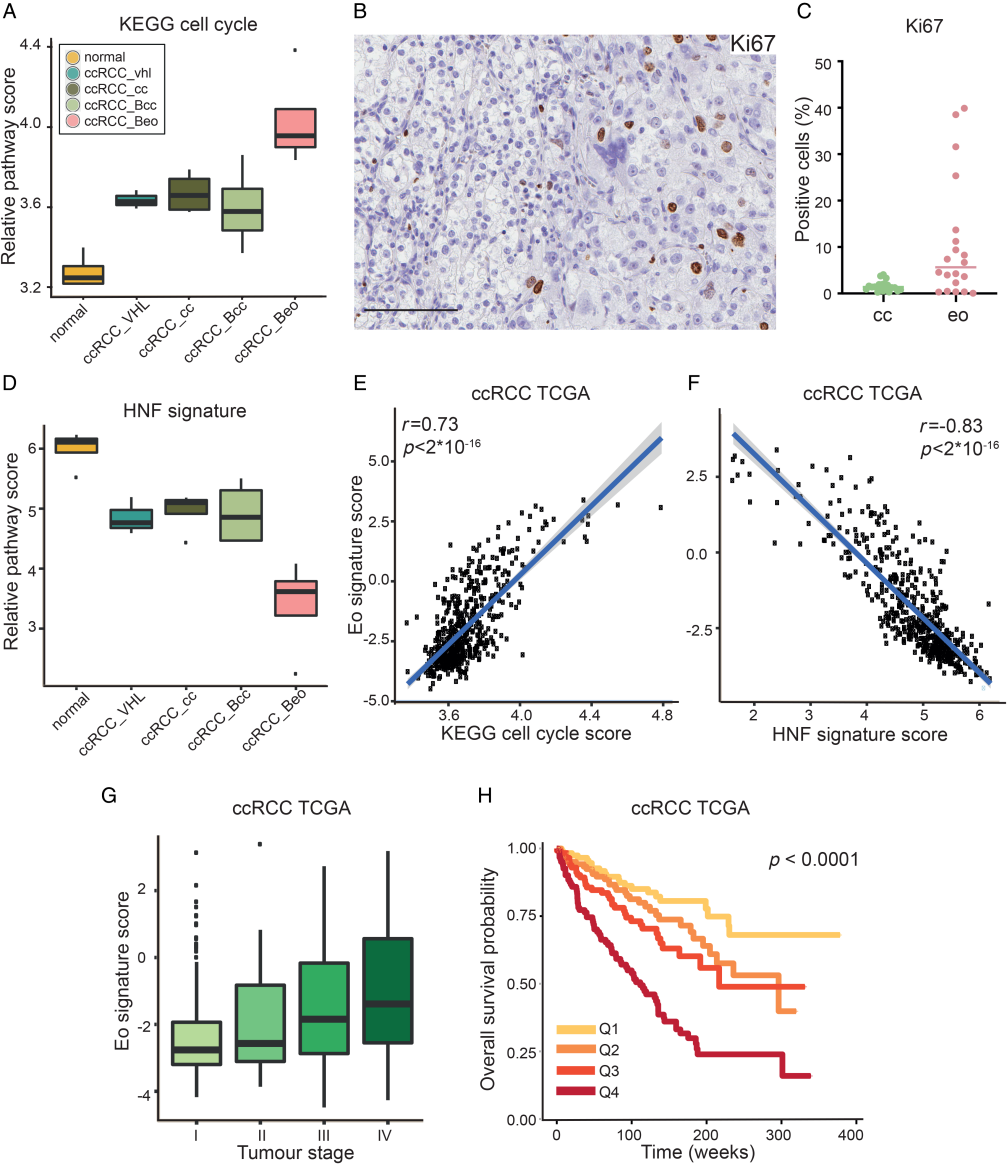
(A) Expression levels of the KEGG cell cycle signature in normal kidney tissue and ccRCCs of different phenotypes. (B) Immunohistochemical staining of biphasic ccRCC for Ki67. Positive nuclei are more abundant in the eosinophilic (right) area. Scale bar = 100 μm. (C) Quantification of % cells positive for Ki67 in the double cores from ten cases of eosinophilic (eo) or clear cell (cc) ccRCC included in the validation cohort. *p* = 0.0032, Student's unpaired *t*‐test. (D) Relative expression of proximal tubule specific HNF‐regulated gene network in sequenced samples. (E, F) Correlation between the eosinophilic gene signature and the KEGG cell cycle signature (E) or HNF signature (F) in ccRCCs from the TCGA sample collection. (G, H) The eosinophilic expression signature is enriched in ccRCCs from the TCGA of higher stage (G) and is associated with worse patient overall survival (H), based on 495 ccRCCs stratified into quartiles based on each tumour's individual eo signature score. *P* value represents a log‐rank test.

Gene set enrichment analysis was next performed on ranked gene lists of the ccRCC_Beo transcriptome. Selected Gene Ontology (GO) terms enriched in eosinophilic (eo_up) or clear cell (eo_down) tissue are shown in Figure 2D. Among the most significantly enriched networks in the eo_up signature, GO terms correlating to inflammation, such as ‘activation of immune response’, ‘leukocyte migration’, and ‘regulation of T cell activation’, were found, but also to proliferation and replication, such as ‘cell division’, ‘hallmark mitotic spindle’, and ‘KEGG cell cycle’ (Figure 2D). The KEGG cell cycle signature was induced in eosinophilic samples compared to all other ccRCCs (Figure 3A).

To corroborate the findings from RNA sequencing on the protein level, biphasic ccRCC was stained by immunohistochemistry. In addition, a set of ccRCC cores with confirmed eosinophilic or clear cell histology was selected from a tissue microarray as a validation cohort used for quantification of the staining. Double cores from ten cases of each histology were included in this set. A higher proliferation in eosinophilic areas was confirmed by significantly more abundant Ki67 staining compared with clear cell areas in biphasic ccRCC (Figure 3B and supplementary material, Figure S2). Quantification of Ki67 positivity in the validation cohort further verified this observation (Figure 3C). ccRCCs are thought to originate from proximal tubular epithelial cells. Gene programs specific for this nephron compartment are conserved in ccRCC. Among these, a hepatocytic nuclear factor (HNF)‐regulated gene network restricted to the proximal tubule has been shown to be retained in lower‐stage ccRCCs but reduced in ccRCCs of higher stage [14]. We found the HNF‐regulated proximal tubule specific gene program to be downregulated in eosinophilic tissue, indicating a lower degree of differentiation (Figure 3D). Furthermore, the GO term ‘hallmark EMT’, was enriched in the eo_up signature, while gene sets correlating to renal cell structure and function, such as ‘HNF1’, ‘brush border’, and ‘basolateral plasma membrane’, were among GO terms in the eo_down gene rank list (Figure 2D).

In accordance with these findings, transcription factor sequence motif analysis using the PASTAA tool (http://trap.molgen.mpg.de/cgi-bin/pastaa.cgi [15]) of genes upregulated in eosinophilic tissue suggested that these are controlled by transcription factors such as NF‐κB [association score (AS) 6.4, *p* < 1 × 10^−5^] and E2F1 (AS 3.6, *p* < 4.1 × 10^−3^), while HNF1a (AS 7.8, *p* < 1 × 10^−6^), HNF4 (AS 3.8, *p* < 3.6 × 10^−3^), and PPARG (AS 5.9, *p* < 4.2 × 10^−5^) were among transcription factors likely to control the downregulated genes.

The eosinophilic transcriptional signature was next evaluated in ccRCCs included in the TCGA sample collection. High eo_up score correlated positively to the KEGG cell cycle signature, and negatively to the HNF signature (Figure 3E,F). Indeed, a high eosinophilic signature score was associated with higher tumour stage and worse overall patient survival (Figure 3G,H).

### The rich vascular network of clear cell tissue is lost in eosinophilic areas

**Figure 4:**
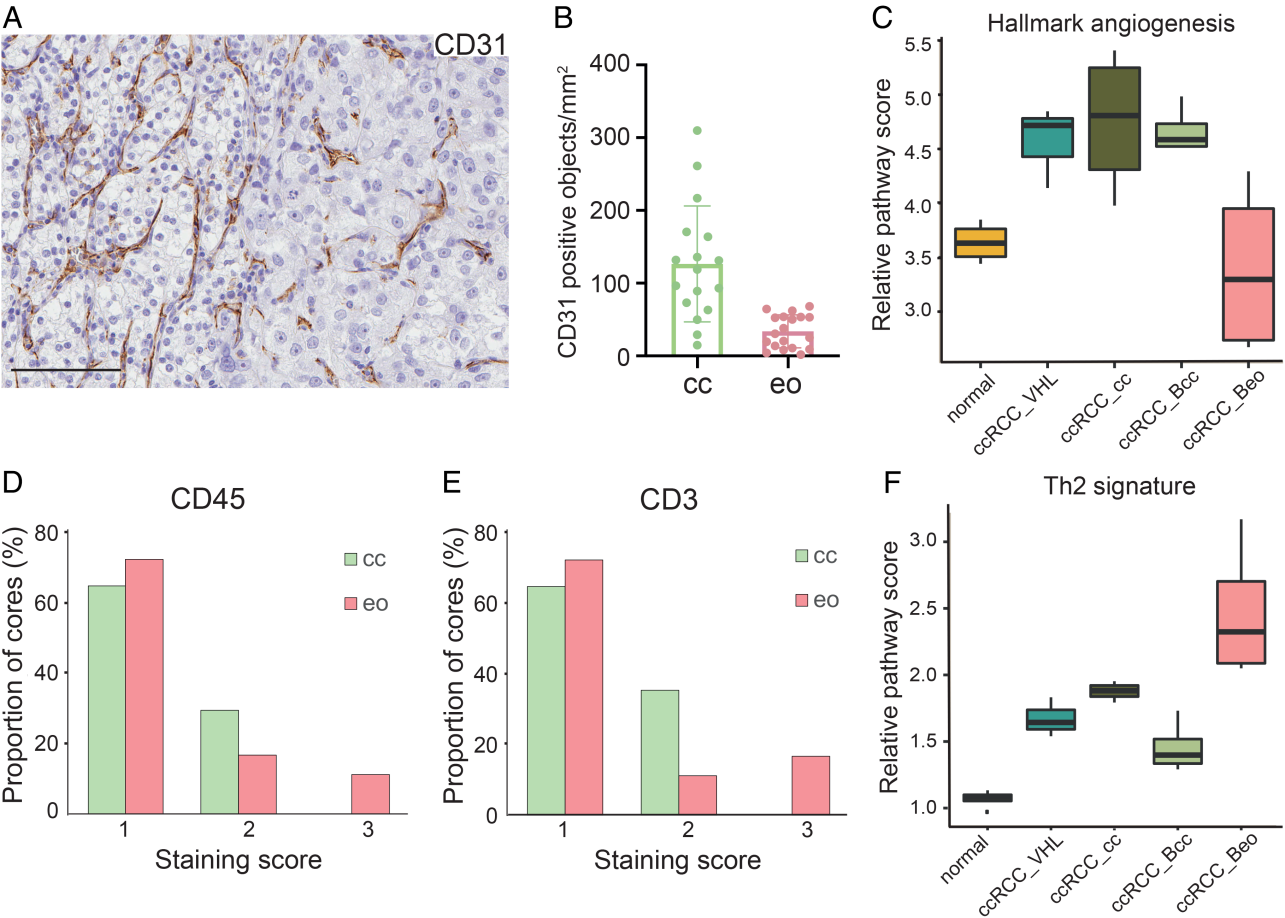
(A) Immunohistochemical staining of biphasic ccRCC tissue for CD31. Note the difference in vessel density between the clear cell (left) and eosinophilic (right) areas. Scale bar = 100 μm. (B) Quantification of CD31‐positive objects per mm^2^ in the validation cohort of eosinophilic and clear cell ccRCCs. *p* < 0.0001, unpaired Student's *t*‐test. (C) Relative expression levels of the ‘hallmark angiogenesis’ gene signature in normal kidney tissue and ccRCCs of different phenotypes as indicated. (D, E) Scoring of CD45 (D) and CD3 (E) staining in ccRCCs of clear or eosinophilic phenotype. Data are presented as % cores for each score, based on evaluation of clear cell or eosinophilic ccRCCs in the validation cohort. Score 1 equals few positive cells and score 3 widespread positivity. *p* < 0.0001 (CD31) and *p* = 0.0008 (CD45), χ^2^ test. (F) Relative expression of the Th2 gene signature in normal kidney and ccRCCs of different phenotypes.

HIF activation results in an enhanced angiogenic drive in ccRCC. Interestingly, the GO terms ‘endothelium development’, ‘blood vessel morphogenesis’, and ‘smooth muscle cell differentiation’ were enriched in the eo_down signature (Figure 2D and supplementary material, Table S4). Staining of biphasic ccRCC for the endothelial cell marker CD31 confirmed that while tissue in clear cell areas displayed the expected dense and intricate vasculature, vessels in eosinophilic tissue were strikingly few (Figure 4A). This pattern was repeated in the tissue used for RNA sequencing (supplementary material, Figure S3A). Indeed, quantification of microvascular density (MVD) in the validation cohort showed a significantly lower MVD in eosinophilic tissue (Figure 4B). As expected, the GO term ‘hallmark angiogenesis’ was enriched in ccRCC_cc, ccRCC_VHL, and ccRCC_Bcc compared with normal kidney, but expression of these genes was reduced in ccRCC_Beo (Figure 4C).

### Immune cells in eosinophilic ccRCC

As mentioned above, among the GO terms enriched in the eosinophilic signature, several were associated with an inflammatory phenotype. To further investigate the immune cell phenotype in ccRCC, the validation cohort was stained for a selection of immune cell markers. Cells positive for the pan‐leukocyte marker CD45 were evenly distributed at low to intermediate levels across clear cell cores, while eosinophilic tissue showed greater variability. Some eosinophilic cores were almost completely negative; in others, the staining was widespread (Figure 4D). No significant differences were found in the number of CD68‐positive monocytic cells, and very few CD20‐positive cells were identified across all analysed cores, indicating a low number of infiltrating B‐cells in ccRCC (supplementary material, Figure S3B). In contrast, widespread positivity for the T‐cell marker CD3 was exclusively found in eosinophilic tissue, and the scoring distribution of CD3‐positive cells was significantly different in eosinophilic compared with clear cell tissue (Figure 4E). The level of T‐cell infiltration correlates to clinical outcome in ccRCC [16], and a gene signature associated with type 2 T‐helper cells identified by Bindea *et al* [17] correlates to poor survival in all types of RCC [1, 16]. We found elevated expression of this gene set in eosinophilic tissue compared with both normal kidney and the other ccRCC samples (Figure 4F).

### The mTOR pathway is activated in eosinophilic ccRCC

**Figure 5:**
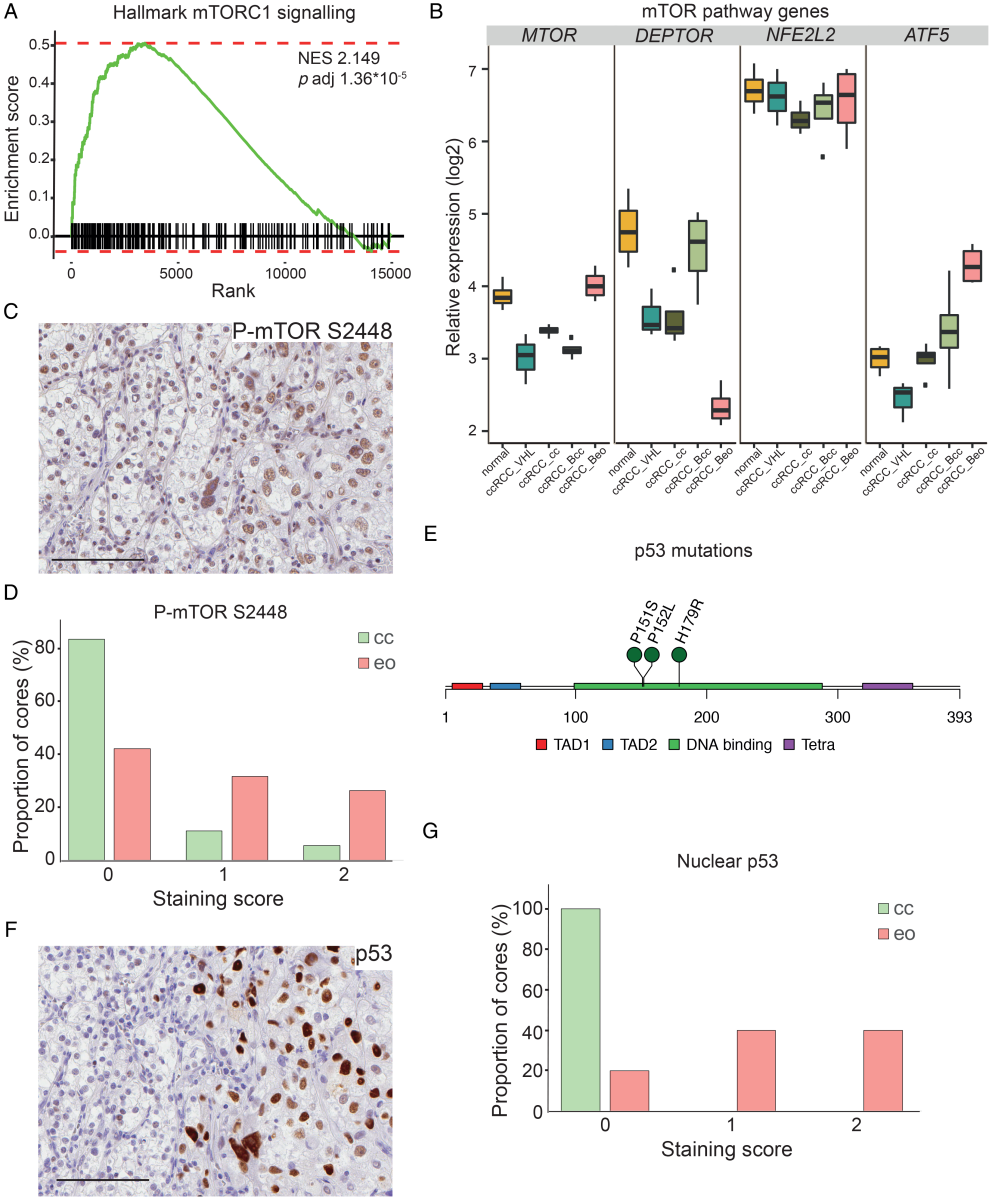
(A) GSEA plot showing enrichment of mTORC1 signalling in the eosinophilic signature. NES, normalized enrichment score. (B) Expression levels of mTOR pathway genes as indicated in normal kidney tissue and ccRCCs of different phenotypes. (C) Immunohistochemical staining of biphasic ccRCC tissue for S2448 phosphorylated mTOR. To the left, clear cell tissue; to the right, eosinophilic. Scale bar = 100 μm. (D) Quantification of S2448 phosphorylated mTOR in the validation cohort of clear cell or eosinophilic ccRCC. Data are presented as % cores for each score. 0 equals no positive cells and 2 equals widespread positivity. *p* < 0.0001, χ^2^ test. (E) Lollipop plot showing localization of *TP53* mutations identified in ccRCC_Beo samples. The mutations were in patient 1, H179R (c.536A>G); in patient 3, P151S (c.451C>T); and in patient 5, P152L (c.455C>T). TAD, transcriptional activation domain; Tetra, tetramerization domain. (F) Immunohistochemical staining for p53 in biphasic ccRCC. Eosinophilic area is to the right and clear cell to the left. Scale bar = 100 μm. (G) Quantification of nuclear p53 in eosinophilic and clear cell ccRCCs in the validation cohort, presented as % cores where staining was negative (0), scattered (1) or widespread (2). *p* < 0.0001, χ^2^ test.

In ccRCC, mTOR is one of the most frequently mutated genes [4], and the mTOR pathway is regularly activated [18, 19]. mTOR exerts its effects as part of two protein complexes, mTOR complex 1 and 2 (mTORC1/2). Activation of mTORC1 is linked to anabolic processes, and this complex is the primary target of rapamycin analogues [20]. We found the GO term ‘hallmark MTORC1 signalling’ to be enriched in eosinophilic tissue (Figures 2D and 5A). In accordance with previous studies of the mTOR protein [21, 22], *MTOR* mRNA was reduced in ccRCC compared with normal kidney (Figure 5B). However, in eosinophilic samples, *MTOR* was increased compared with the ccRCC samples. In addition, *DEPTOR* (DEP domain‐containing mTOR‐interacting protein), a negative regulator of mTORC1/2 that is reduced in ccRCC compared with normal kidney, was further downregulated in eosinophilic tissue (Figure 5B). Immunohistochemical staining of biphasic ccRCC showed no difference in phosphorylation of mTOR at S2481 (data not shown), associated with active mTORC2. In contrast, phosphorylation at S2448, correlating to mTORC1 activity [23], was induced in eosinophilic areas (Figure 5C). Quantification of phosphorylated S2448 in the validation cohort confirmed this observation (Figure 5D). This pattern was repeated in ccRCC_Bcc and ccRCC_Beo samples used for RNA sequencing (supplementary material, Figure S3C).

Most publications regarding mTOR focus on protein activity; fewer concern its transcriptional regulation. However, NRF2 and ATF5 are suggested to regulate the expression of mTOR [24, 25]. We found no change in the mRNA levels for NRF2 (*NFE2L2*) in ccRCC_Beo compared with ccRCC_Bcc samples, but *ATF5* was induced, posing a possible explanation for increased mTOR mRNA in eosinophilic ccRCC (Figure 5B).

### *TP53* mutations in eosinophilic ccRCC

*TP53* is the most frequently mutated gene in human cancer [26], yet in ccRCC, *TP53* mutations are considered to be rare. The TCGA report from 2018 [1] reported *TP53* mutations in 2.6% of ccRCCs. By performing mutation calling analysis on the RNA‐sequencing data, we identified missense mutations in *TP53* in three of four eosinophilic samples. The mutations were all within the DNA binding domain of *TP53* and are reported to result in loss of function (Figure 5E) (https://cancer.sanger.ac.uk/cosmic). Variations in *TP53* were not found in any other sample, either in the corresponding clear cell tissue from biphasic tumours or in the other ccRCCs or normal kidney samples.

Nuclear p53 positivity is considered a sign of mutated protein, as mutations tend to stabilize the protein, resulting in nuclear accumulation [27]. Nuclear p53 was confirmed in the eosinophilic samples carrying missense mutations (supplementary material, Figure S4). Eosinophilic tissue from patient 2, where no mutations were found, also displayed cells with nuclear p53, although at lower frequency. Accordingly, accumulation of p53 seems to be restricted to eosinophilic areas in biphasic ccRCC (Figure 5F). Nuclear p53 was detected in eight of the ten stained eosinophilic ccRCCs included in the validation cohort; in four of these, the positivity was widespread. In contrast, all cores from the ten ccRCCs of clear cell histology were negative (Figure 5G). These results suggest that mutated *TP53* is associated with the eosinophilic phenotype.

### Cytoplasmic lipid content is decreased in eosinophilic ccRCC

**Figure 6:**
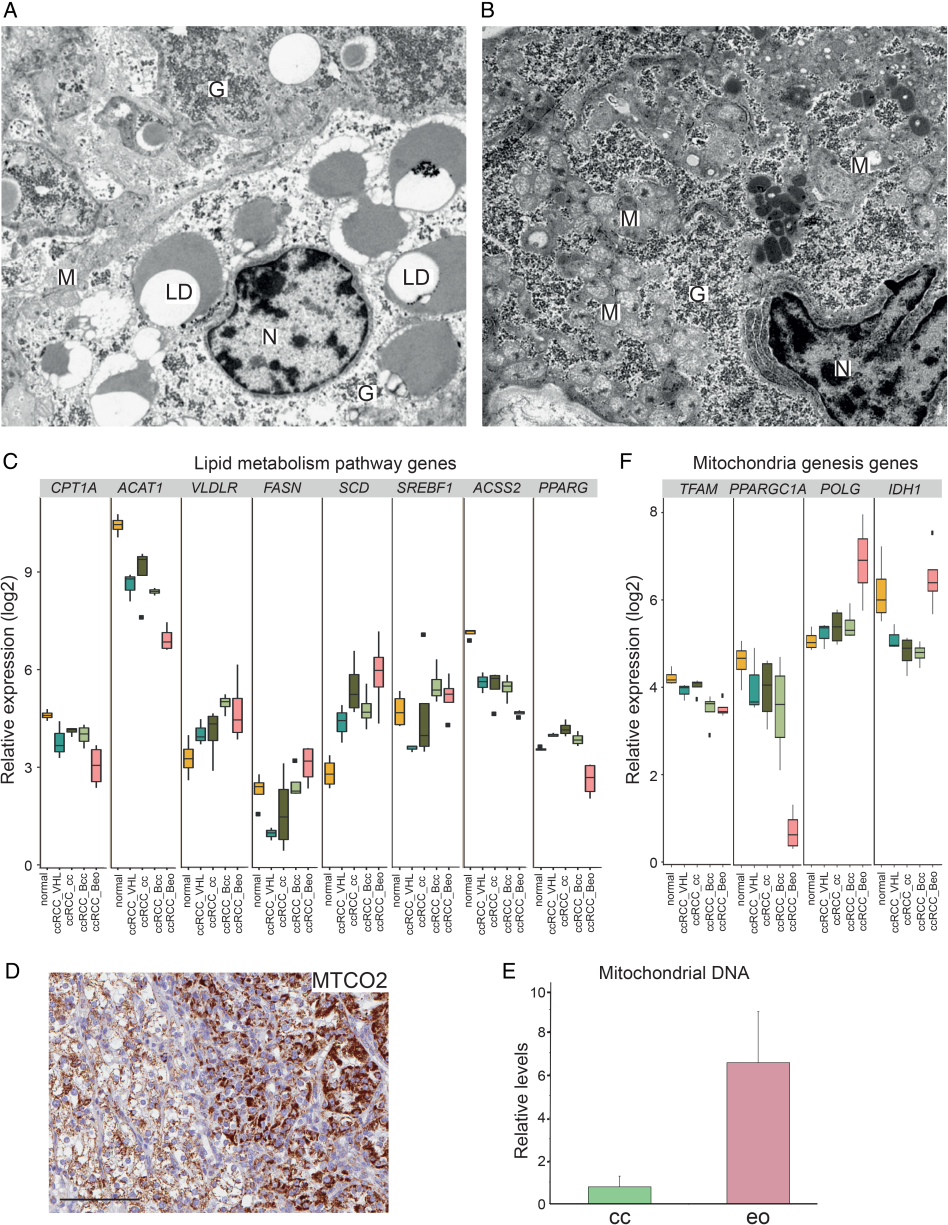
(A, B) Electron microscopy images of clear cell (A) or eosinophilic area (B) in a case of biphasic ccRCC. G, glycogen granules; LD, lipid droplets; M, mitochondria; N, nucleus. (C) Relative RNA levels of genes associated with lipid metabolism in normal kidney cortex and ccRCC samples of different phenotypes as indicated. (D) Immunohistochemical staining for MTCO2 in biphasic ccRCC, where the eosinophilic area is to the right. Scale bar = 100 μm. (E) Relative mitochondrial DNA levels in clear cell or eosinophilic tissue from biphasic ccRCC. *n* = 2. (F) Relative expression of the indicated genes in normal kidney and ccRCCs of different phenotypes.

The most striking histological difference between clear cell and eosinophilic ccRCC concerns cytoplasmic content. While the cytoplasm of clear cells appears empty, eosinophilic tissue displays a pink granular cytoplasm in H&E sections. Early electron microscopy (EM) studies reported the cytoplasm of ccRCC to be filled with glycogen and lipid droplets, with sparse organelles [28]. Granular or eosinophilic RCC, in contrast, was reported to contain little glycogen or lipids; instead, the number of mitochondria and ribosomes was increased [12, 28]. To confirm these findings, and connect ultrastructural imaging to the eosinophilic phenotype, we performed EM on biphasic ccRCC (Figure 6A,B). Indeed, in clear cell areas, cells were filled with lipid droplets, while mitochondria were scarce. No lipid droplets were found in eosinophilic cells; however, glycogen granules were abundant.

Several mechanisms are suggested for the cytoplasmic lipid accumulation in ccRCC. HIF‐induced VLDLR (very‐low‐density lipoprotein receptor) increases the uptake of extracellular lipids [29], and genes involved in fatty acid (FA) and lipid synthesis such as *FASN* (FA synthase) and *SCD* (stearoyl‐CoA desaturase 1) are induced [30, 31]. In addition, lipid degradation seems to be unbalanced. CPT1A (carnitine palmitoyltransferase 1A) is required for transport of FAs into mitochondria, where they undergo degradation by β‐oxidation. In ccRCC, *CPT1A* is downregulated by HIFs, resulting in reduced mitochondrial β‐oxidation [32].

Decreased eosinophilic lipid content indicates rewired lipid metabolism compared with clear cell ccRCC. Among GO terms enriched in the eo_down signature, ‘hallmark adipogenesis’ and ‘hallmark fatty acid metabolism’ were found (Figure 2D). Genes in these GO terms are mainly connected to β‐oxidation, but also lipid synthesis. Accordingly, we found the compared to normal kidney, low expression of *CPT1A* in ccRCC_Bcc samples to be further reduced in eosinophilic tissue. *ACAT1* (acetyl‐coenzyme A acetyltransferase 1a), encoding an enzyme involved in β‐oxidation, was also reduced in eosinophilic tissue, indicating an even lower capacity for FA degradation (Figure 6C). Thus, increased degradation does not seem to explain the reduced eosinophilic lipid content. *VLDLR*, *FASN*, and *SCD* remained at least equally high in eosinophilic as in clear cell samples (Figure 6C). *PPARG* and *SREBF1* encode transcriptional regulators of lipogenesis [33, 34]. RNA levels of *SREBF1* did not differ in eosinophilic samples compared with clear cell, but *ACSS2*, a target gene of *SREBF1*, was expressed at very low levels (Figure 6C). In addition, *PPARG* expression was strongly reduced in ccRCC_Beo samples (Figure 6C), and the GO term ‘PPAR signalling pathway’ was significantly downregulated (Figure 2D), suggesting that lipid content is reduced in eosinophilic tissue through transcriptional regulation of lipogenesis.

### Mitochondria in eosinophilic ccRCC

As mentioned, in contrast to clear cells, granular RCC is suggested to harbour more mitochondria. Immunohistochemical staining for mitochondrially encoded cytochrome C oxidase II (MTCO2) in biphasic ccRCC showed strong positivity in eosinophilic areas (Figure 6D). Quantification of mitochondrial DNA in eosinophilic compared with clear cell tissue from two biphasic ccRCCs supported this notion (Figure 6E). EM imaging of biphasic ccRCC further corroborated the observation of more abundant mitochondria in eosinophilic areas (Figure 6A).

Intriguingly, the RNA‐seq data did not reveal any convincing explanation of the mechanism behind this apparent shift in mitochondrial load. The mitochondrial transcription factor *TFAM* was expressed at similarly low levels in ccRCC_Beo and ccRCC_Bcc samples (Figure 6F). In addition, expression of *PPARGC1A* (peroxisome proliferator‐activated receptor gamma coactivator 1‐alpha), a master regulator of mitochondrial biogenesis [35], was reduced in eosinophilic compared with clear cell samples (Figure 6F). Analysis of genes listed in the human MitoCarta [36, 37] in ccRCC_Beo and ccRCC_Bcc samples revealed that a majority of differentially expressed genes were downregulated in eosinophilic ccRCC. The downregulated genes were associated with metabolic functions such as TCA cycle and FA β‐oxidation. Indeed, ‘hallmark oxidative phosphorylation’ was among the significantly downregulated GO terms in eosinophilic samples (Figure 2D). Genes induced in eosinophilic tissue were associated with mitochondrial ribosomes and translation; among these was *POLG* (DNA polymerase subunit gamma), encoding the polymerase used for mitochondrial DNA replication (Figure 6F).

EM imaging of biphasic ccRCC confirmed the observation of more abundant mitochondria in eosinophilic areas, although the mitochondria appeared to be small and with incompletely formed cristae (Figure 6A), as has been reported by Tickoo *et al* [38]. Cells with defective mitochondria can utilize glutamine‐dependent reductive carboxylation to obtain acetyl‐CoA to support growth and proliferation [39]. Cytosolic conversion of glutamine‐derived α‐ketoglutarate to citrate by isocitrate dehydrogenase (*IDH1*) provides an alternative source of acetyl‐CoA when glucose levels are limited, or TCA cycling is defective. Increased reductive carboxylation, glutamine dependency, and uptake have been shown in ccRCC cell lines [40, 41]. Interestingly, *IDH1* was highly expressed in eosinophilic tissue, indicating a possible role for reductive carboxylation in these cells (Figure 6D). Together, these observations raise questions regarding the biogenesis and metabolic capacity of mitochondria in eosinophilic tissue, and further studies are required to untangle the mechanism behind the increased amounts found in eosinophilic cells.

## Discussion

The heterogeneity of ccRCC complicates the connection of large‐scale data to histological phenotypes. In this study, we have compared for the first time the transcriptomes of isolated and histologically confirmed eosinophilic and clear cell components from ccRCC with both patterns. Although based on a small number of cases, the findings from RNA‐sequencing data were validated in the TCGA data set, and by immunohistochemical staining of an additional ccRCC cohort. The eosinophilic transcriptome indicated a lower degree of differentiation combined with increased proliferative drive, confirmed by immunohistochemical staining for Ki67. In concordance, the eosinophilic gene signature was associated with tumours of higher stage and worse outcome in ccRCCs of TCGA.

We further found that while clear cell tissue displayed an elaborate, dense microvasculature, eosinophilic areas were significantly less vascularized. One possible explanation could be that eosinophilic cells outgrow neovascularization due to increased proliferation. Interestingly, the GO term ‘hallmark hypoxia’ was enriched in eosinophilic samples (Figure 2D). Association between MVD and tumour grade is controversial in ccRCC [42, 43]. Our findings of decreased MVD in eosinophilic tissue together with a correlation of this phenotype to higher stage support an association of high MVD with better prognosis. Although the vasculature was more extensive in clear cell tissue, eosinophilic areas appeared more inflammatory with more profuse infiltration of T cells. A type 2 T‐helper (Th2) cell signature associated with poor prognosis was indeed enriched in eosinophilic samples.

Moreover, we found the mTOR pathway, specifically the mTORC1 complex, to be preferentially activated in eosinophilic tissue based on RNA expression and histological S2448 phosphorylation. This is interesting in light of a recent publication where elevated phosphorylation of mTOR at S2448 was found in metastatic RCC [21]. This could indicate that eosinophilic cells have a higher propensity to metastasize. In this context, it would be interesting to investigate whether the presence of eosinophilic cells impacts the response to mTOR inhibition. Two rapamycin analogues, everolimus and temsirolimus, are approved for treatment of metastatic RCC; however, the response rate varies, and these inhibitors are therefore not considered first‐line treatments. Improved stratification of patients could potentially increase the response rate; several studies have suggested that patients with activated mTOR are more likely to respond [44, 45, 46].

*TP53* mutations are considered rare in ccRCC but are more frequently found in other subtypes of RCC, including papillary and chromophobe. Yet positive immunohistochemical staining of p53 significantly correlates to poor prognosis only in ccRCC [47], and is an independent predictor of recurrence following nephrectomy [48]. Gerlinger *et al* detected *TP53* mutations in 6% of ccRCC cases when analysing single biopsies, while multiregional sequencing showed mutations in 40% [49]. This indicates the presence of focal areas with mutated *TP53*, easily missed when analysing single biopsies. We found *TP53* mutations in three of four eosinophilic samples, and nuclear p53 staining in eight of ten analysed eosinophilic cores. Together, these results suggest that mutated *TP53* is associated with the eosinophilic phenotype and that it is focally more prevalent in ccRCC than previously appreciated, probably underestimated due to intratumor heterogeneity.

Cytoplasmic lipids and glycogen are suggested to decrease with stage in ccRCC [50]. Indeed, EM imaging showed that lipid content was reduced in eosinophilic compared with clear cell tissue. Decreased expression of the *PPARG* gene program suggests limited lipogenesis. However, although eosinophilic tissue harboured numerous mitochondria, transcriptional programs associated with mitochondrial biogenesis and metabolism were downregulated. In addition, the mitochondria appeared malformed. Upregulation of *IDH1* indicates a role for reductive carboxylation of glutamine. It is interesting to note that the metabolic phenotype of eosinophilic ccRCC resembles that of established RCC cell lines. We have previously shown an elevated mitochondrial load in RCC cell lines compared with cultured primary clear cells [11]. RCC cell lines are dependent on glutamine and perform reductive carboxylation. In addition, most RCC cell lines do not display cytoplasmic lipid accumulation to the same extent as cultured primary clear cells.

It is known that ccRCC is histologically heterogeneous. Increased knowledge regarding different biological potentials in various ccRCC phenotypes could improve predictions of treatment response. The findings presented here could imply that clear cell ccRCCs should respond to anti‐angiogenic and glycolytic inhibitors, while ccRCCs with eosinophilic component might benefit from mTOR or glutaminase inhibition. Furthermore, an increased inflammatory signature in eosinophilic areas suggests implications for response to immune therapies. It might also be a sign of increased formation of neoantigens, since eosinophilic tissue areas associate with *TP53* mutations. We estimate that a predominantly eosinophilic histology is seen in at least 20% of ccRCCs and that this percentage increases with stage and grade. A possible impact on tumour characteristics and clinical behaviour of this histological pattern therefore warrants further study.

## Author contributions statement

HN and MEJ conceived experiments and collected material. HN carried out experiments. DL and HA guided and performed bioinformatic analysis. CB and LHS carried out RNA mutation calling analysis. JL performed electron microscopy imaging. All the authors contributed to the interpretation of the data, were involved in writing the paper, and had final approval of the submitted version of the manuscript.

## Supporting information


**Supplementary materials and methods**

**Figure S1.** H&E staining showing the histology of clear cell and eosinophilic tumour samples, respectively, from the five biphasic ccRCCs selected for RNA sequencing
**Figure S2.** Immunohistochemical staining for Ki67 in clear cell and eosinophilic ccRCC samples, respectively, selected for RNA sequencing
**Figure S3.** Characterization of clear cell and eosinophilic tissue used for RNA sequencing
**Figure S4.** Staining for p53 in clear cell and eosinophilic tissue used for RNA sequencing

**Table S1.** Patient information related to tissue used for RNA sequencing

**Table S2.** Antibodies used for immunohistochemical staining

**Table S3.** List of 174 significantly differentially expressed genes in ccRCC_Beo compared with ccRCC_Bcc

**Table S4.** List of GO terms associated with the eo signature

**Table S5.** RNA‐sequencing expression data

## Data Availability

RNA‐sequencing data are available in supplementary material, Table S5.
