# Supplementary material for: Features of increased malignancy in eosinophilic clear cell renal cell carcinoma
Source: J Pathol. 2020 Sep 24;252(4):384–97. doi: 10.1002/path.5532 (PMC7756750; doi:10.1002/path.5532)
Supplement: Supplementary file 1 — Supplementary materials and methods [file PATH-252-384-s001.docx]

**Features of increased malignancy in eosinophilic clear cell renal cell carcinoma**

H Nilsson *et al. J Pathol* DOI: 10.1002/path.5532

**Supplementary materials and methods**

Reference numbers refer to the main text list

*RNA isolation, sequencing, and TCGA data analysis*

After overnight incubation at 4 °C, tissue collected in RNAlater was disrupted in TRIzol Reagent (Ambion, Thermo Fisher Scientific, Waltham, MA, USA) using a Tissue Lyzer LT (Qiagen, Hilden, Germany). After chloroform phase separation, RNA was precipitated with 70% EtOH and washed and eluted on RNeasy mini columns from the RNeasy mini kit (Qiagen). RNA integrity was determined using the RNA 6000 Nano kit on a Bioanalyzer 2100 (Agilent, Santa Clara, CA, USA).

Library preparation and RNA sequencing were performed at the National Genomics Infrastructure facility SciLifeLab in Stockholm, Sweden. Strand-specific, single-end sequencing libraries were prepared using the Illumina TruSeq protocol and poly-A selection. Libraries were sequenced on an Illumina HiSeq 2500 instrument using Rapid Mode 1×50bp across four lanes, giving >20 M reads per sample. Reads were aligned to the GRCh37 human reference genome using TopHat2 2.0.4 and RPKM/FPKM values were calculated using Cufflinks 2.1.1. All but one sample (R117 ccRCC_VHL) passed the initial quality control of the sequencing results and were selected for further analysis. Gene level FPKM values obtained from Cufflinks were log_2_-transformed upon adding an offset of +1 and analysed using the R statistical language (version 3.6.1). Hierarchical cluster analyses (HCAs) and principal component analyses (PCAs) were performed using the *hclust* and *prcomp* functions within the *stats* R-package. For HCA, 1 − Pearson correlation was used as the distance measure and Ward’s method was used for agglomeration. Differential gene expression between samples from clear cell and eosinophilic parts of the patients’ tumours was determined using paired limma analysis [51]. Up- and down-regulated genes to define the gene signature of eosinophilic growth (eo up) were selected from the limma analysis using adjusted *p* < 0.05. Sample-wise gene signature scores were calculated as the mean expression value of all genes included in the respective gene signature. Expression values of genes with negative regulation (downregulated) were weighted with −1. Gene set enrichment analysis (GSEA) was performed using the *fgsea* package in R [52] and the MSigDBv7.0 collection of gene sets (http://[broad.mit.edu/gsea/msigdb](http://broad.mit.edu/gsea/msigdb%22%20%5Ct%20%22_blank)). Here, the limma results were used and genes were ranked according to their relative log_2_-fold difference between the eosinophilic and the clear cell component.

The Cancer Genome Atlas (TCGA) tumour RNA-seq gene expression data were downloaded from the NCI Genomic Data Commons (GDC) database (https://gdc.cancer.gov). Log_2_-transformed upper quartile normalized FPKM values were used after adding an offset of +1. Clear cell carcinoma RCC TCGA samples were selected from the KIRC, KICH, and KIRP projects based on the ccRCC molecular subgroups described by Chen *et al* [53]. Survival analysis was performed using the *survival* and *survminer* R-packages. TCGA ccRCC samples were here stratified into quartiles based each tumour’s individual eo score.

*Mutation calling from RNA-seq data*

RNA-seq mutation calling was performed in a similar manner to that described previously [10]. Using a Snakemake workflow [54] and Bioconda packages [55], reads were aligned to the human reference genome GRChg38.p8 (with decoys and additional contigs, patched with dbSNP build 147) using HISAT 2.1.0 [56], and duplicates were marked using biobambam 2.0.97 [57]. Variants were called using VarDict-Java 1.6.0 [58] and parameters -f 0.02 (min 2% variant allele frequency), -r 2 (min 2 reads supporting variant allele), and -q 20 (min read quality 20). Variants were annotated with a variety of data sources such as the Catalogue of Somatic Mutations in Cancer (COSMIC) v87 [59,60] using vcfanno [61], and variant effects on canonical transcripts were predicted using SnpEff [62]. Variants were filtered as described previously [10]; importantly, germline events were filtered using dbSNP build 151 [63], the Genome Aggregation Database (gnomAD) [64], SweGen version 20171025 [65], and the Danish Genome Project population reference [66], and RNA editing events were filtered using a variety of databases [67–70]. All filters were applied using SnpSift [71].

*Electron microscopy*

Cancer tissue was sampled from macroscopically probable clear cell and eosinophilic areas. Pieces measuring about 2 mm were prepared for light and electron microscopy (EM), respectively. For EM, samples were fixed for 5 days in 2% paraformaldehyde and 2% glutaraldehyde in Millonig’s phosphate buffer (pH 7.4) and dehydrated in ethanol followed by propylene oxide. Infiltration in AGAR 100 resin (Agar Scientific Ltd, Stansted, UK) was performed and followed by polymerization for 48 h at 60 °C. Sections cut at 70 nm were contrasted with uranyl acetate and lead citrate. Light microscopically confirmed clear and eosinophilic ccRCC areas were photographed using a Tecnai G2 Spirit (FEI Company, Hillsboro, OR, USA).
