# Supplementary material for: Features of increased malignancy in eosinophilic clear cell renal cell carcinoma
Source: J Pathol. 2020 Sep 24;252(4):384–97. doi: 10.1002/path.5532 (PMC7756750; doi:10.1002/path.5532)
Supplement: Supplementary file 2 — Figure S1. H&E staining showing the histology of clear cell and eosinophilic tumour samples, respectively, from the five biphasic ccRCCs selected for RNA sequencing Figure S2. Immunohistochemical staining for Ki67 in clear cell and eosinophilic ccRCC samples, respectively, selected for RNA sequencing Figure S3. Characterization of clear cell and eosinophilic tissue used for RNA sequencing Figure S4. Staining for p53 in clear cell and eosinophilic tissue used for RNA sequencing [file PATH-252-384-s002.docx]

**Features of increased malignancy in eosinophilic clear cell renal cell carcinoma**

H Nilsson *et al. J Pathol* DOI: 10.1002/path.5532

**Supplementary Figures S1–S4**


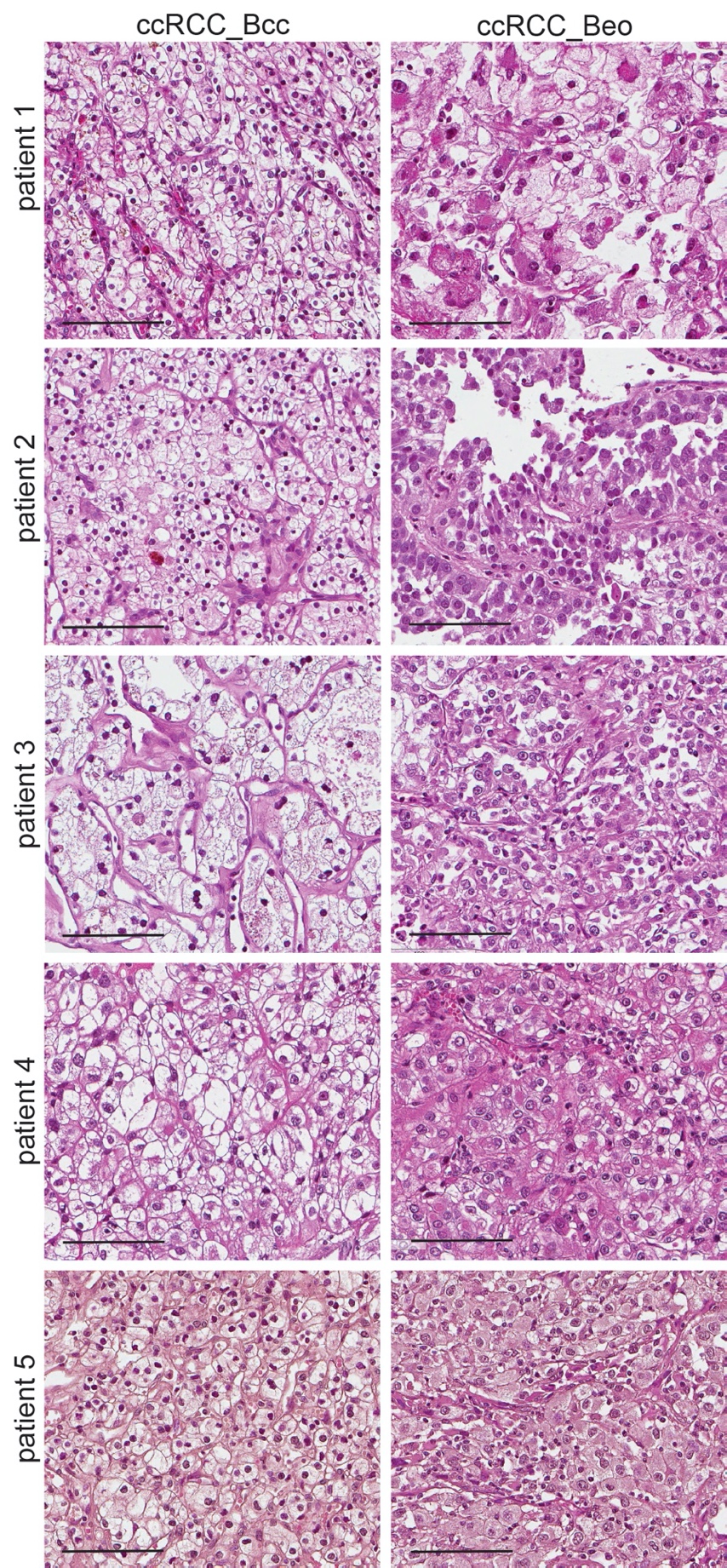


**Figure S1. H&E staining showing the histology of clear cell and eosinophilic tumour samples, respectively, from the five biphasic ccRCCs selected for RNA sequencing.** Scale bars = 100 μm.


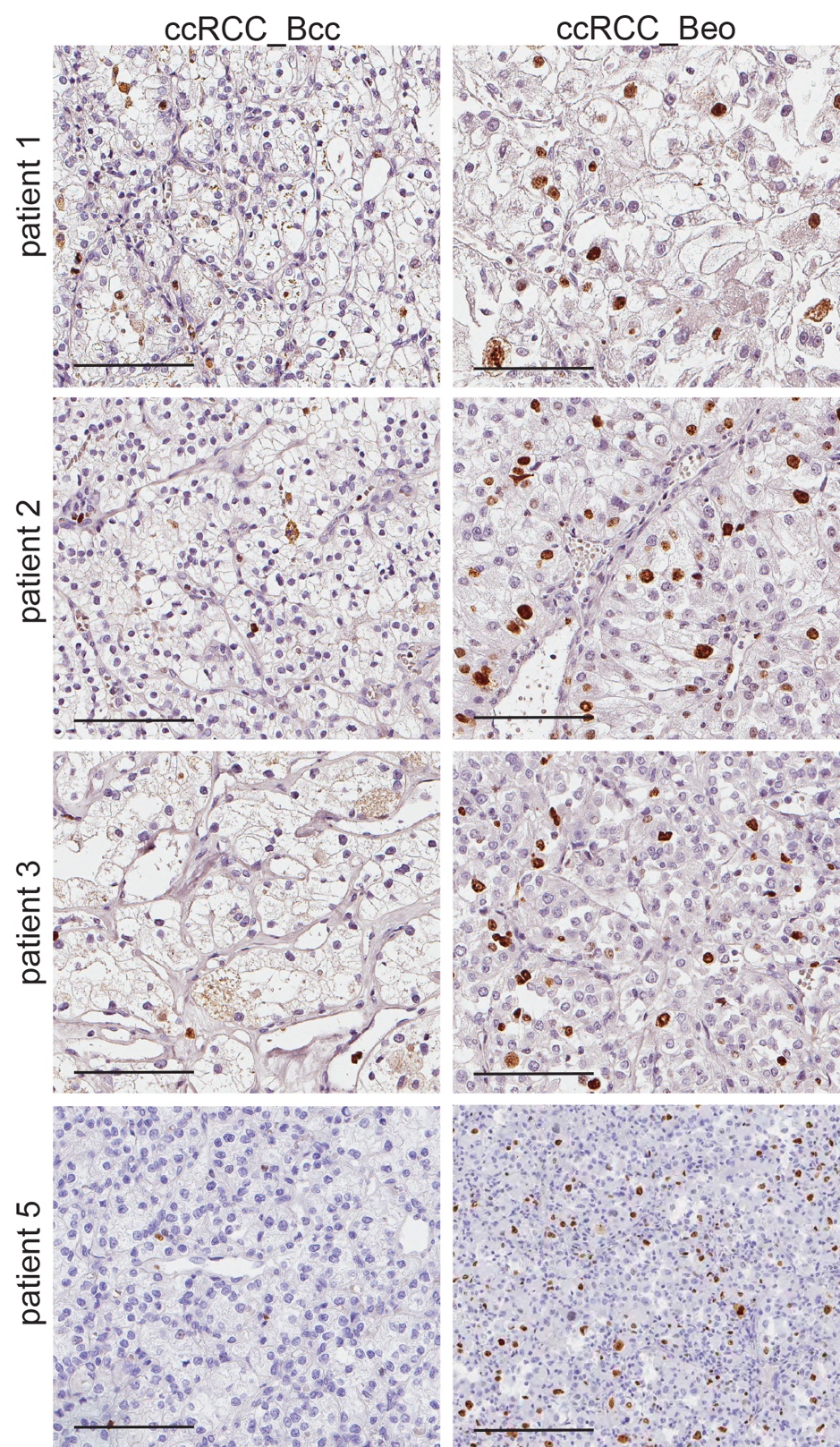


**Figure S2. Immunohistochemical staining for Ki67 in clear cell and eosinophilic ccRCC samples, respectively, selected for RNA sequencing.** Scale bars = 100 μm.


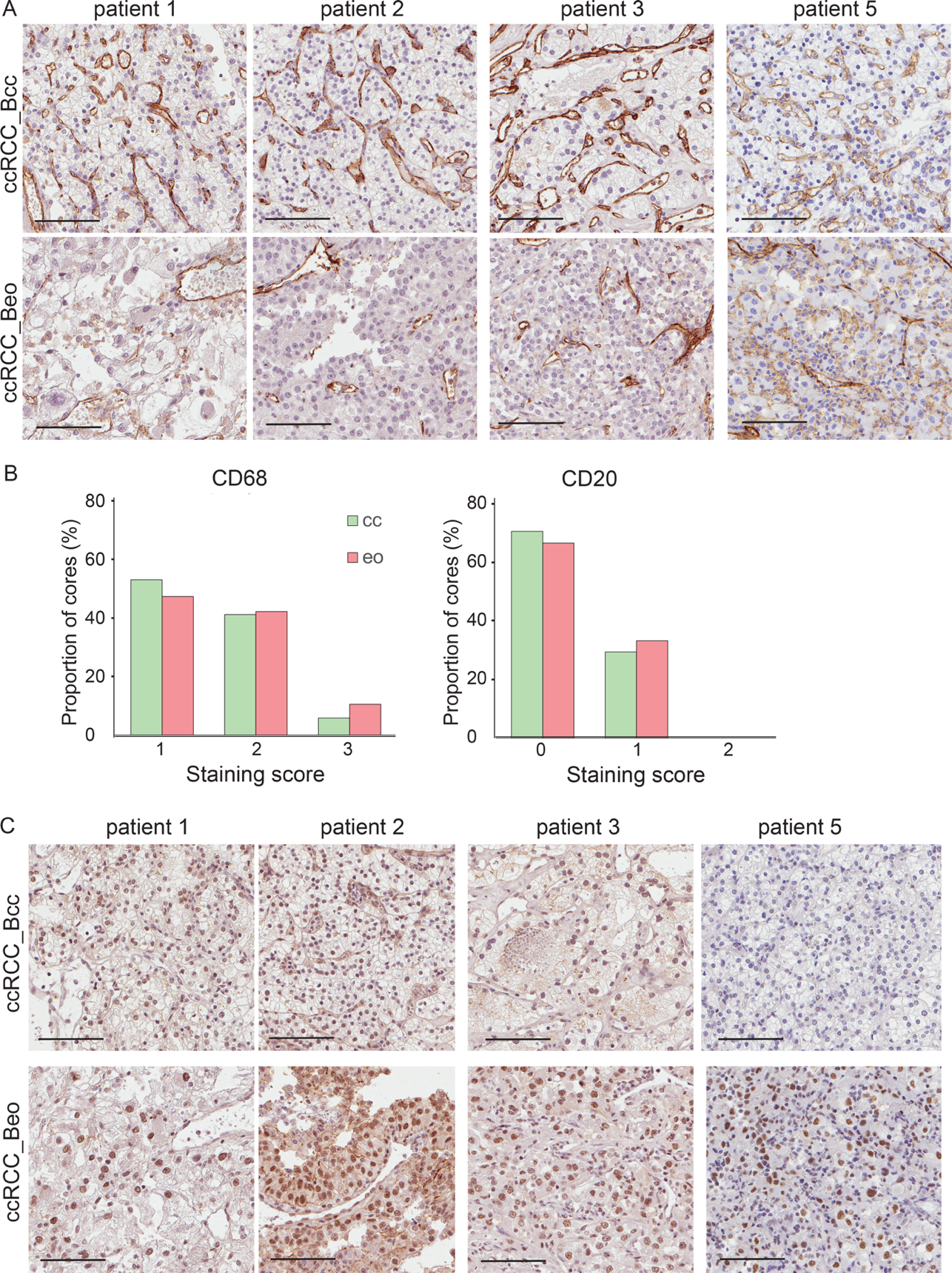


**Figure S3. Characterization of clear cell and eosinophilic tissue used for RNA sequencing.** (A) CD31 staining of clear cell and eosinophilic tissue used for RNA sequencing. Scale bars = 100 μm. (B) Quantification of infiltrating CD68- and CD20-positive cells. Data are presented as % cores for each score, based on evaluation of clear cell or eosinophilic ccRCCs in the validation cohort. Score 0 equals no positive cells and score 3 widespread positivity. *p* = 0.398 (CD68) and *p* = 0.374 (CD20), χ^2^ test. (C) Immunohistochemical staining for mTOR phosphorylated at S2448 in the clear cell and eosinophilic tissue used for RNA sequencing. Scale bars = 100 μm.


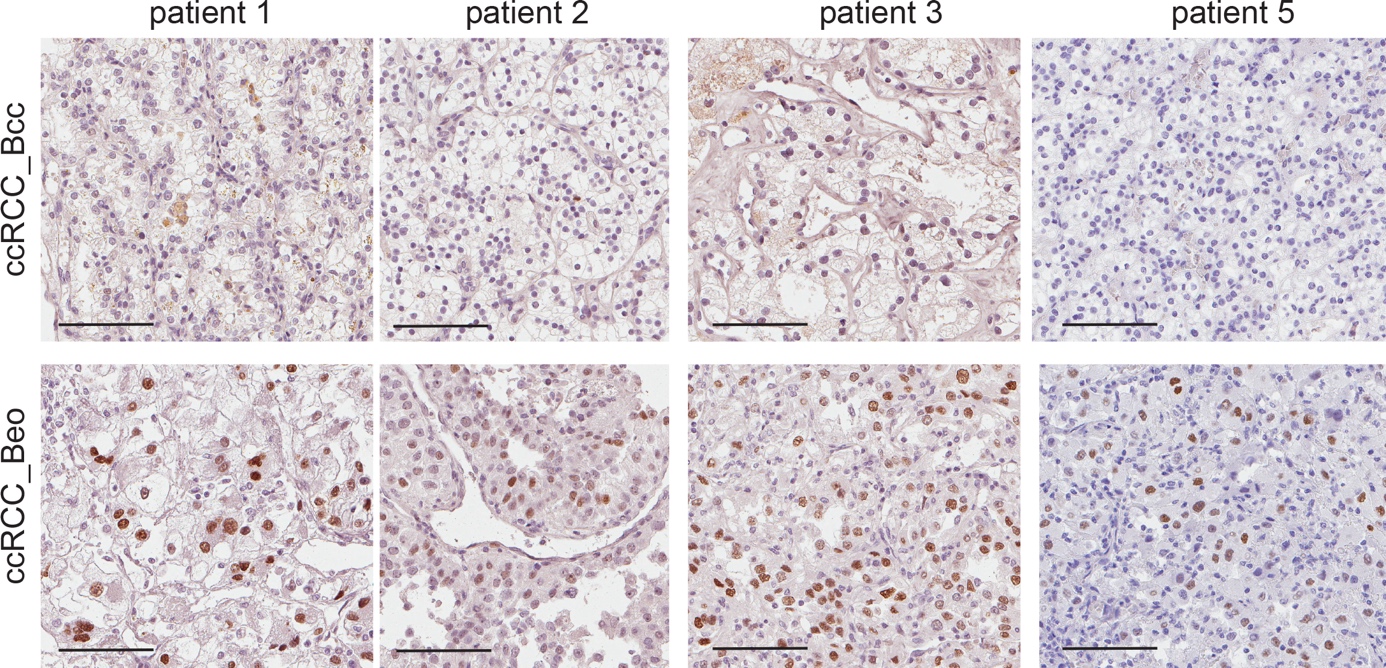


**Figure S4. Staining for p53 in clear cell and eosinophilic tissue used for RNA sequencing.** Scale bars = 100 μm.
