# Supplementary material for: Features of increased malignancy in eosinophilic clear cell renal cell carcinoma
Source: J Pathol. 2020 Sep 24;252(4):384–97. doi: 10.1002/path.5532 (PMC7756750; doi:10.1002/path.5532)
Supplement: Supplementary file 3 — Table S1. Patient information related to tissue used for RNA sequencing [file PATH-252-384-s003.docx]

| **Sample ID** | **Patient No** | **Sex** | **WHO/ISUP grade** | **Stage** | **Phenotype** |
| --- | --- | --- | --- | --- | --- |
| P01_Beo | 1 | F | 4 | pT3a | ccRCC_Beo |
| P01_Bcc | 1 |  |  |  | ccRCC_Bcc |
| P02_Beo | 2 | M | 3 | pT3a | ccRCC_Beo |
| P02_Bcc | 2 |  |  |  | ccRCC_Bcc |
| P03_Beo | 3 | M | 4 | pT3b | ccRCC_Beo |
| P03_Bcc | 3 |  |  |  | ccRCC_Bcc |
| P04_Beo | 4* | F | 3 | pT3a | ccRCC_Beo |
| P04_Bcc | 4* |  |  |  | ccRCC_Bcc |
| P05_Beo | 5 | F | 3 | pT3 | ccRCC_Beo |
| P05_Bcc | 5 |  |  |  | ccRCC_Bcc |
| P06_cc | 6 | F | 2 | pT3a | ccRCC_cc |
| P07_cc | 7 | F | 2 | pT1b | ccRCC_cc |
| P08_cc | 8 | M | 2 | pT3 | ccRCC_cc |
| P09_cc | 9 | F | 2 | pT3a | ccRCC_cc |
| P10_VHL | 10 | M | 1 | pT1a | ccRCC_VHL |
| P12_VHL | 12 | M | 2 | pT1b | ccRCC_VHL |
| P11_VHL | 11 | M | 2 | pT1a | ccRCC_VHL |
| P13_N | 13 | M |  |  | Normal |
| P06_N | 6 | F |  |  | Normal |
| P14_N | 14 | M |  |  | Normal |
| P15_N | 15 | M |  |  | Normal |

**Features of increased malignancy in eosinophilic clear cell renal cell carcinoma**

H Nilsson *et al. J Pathol* DOI: 10.1002/path.5532

**Table S1.** Patient information related to tissue used for RNA sequencing

*ccRCC_Bcc and ccRCC_Beo from patient 4 clustered closer together to each other than to the other samples from their respective phenotype and were not included in the subsequent analyses.
