# Supplementary material for: Features of increased malignancy in eosinophilic clear cell renal cell carcinoma
Source: J Pathol. 2020 Sep 24;252(4):384–97. doi: 10.1002/path.5532 (PMC7756750; doi:10.1002/path.5532)
Supplement: Supplementary file 4 — Table S2. Antibodies used for immunohistochemical staining [file PATH-252-384-s004.docx]

**Features of increased malignancy in eosinophilic clear cell renal cell carcinoma**

H Nilsson *et al. J Pathol* DOI: 10.1002/path.5532

**Table S2.** Antibodies used for immunohistochemical staining

| **Target** | **Clone/cat. No.** | **Supplier** | **Species** |
| --- | --- | --- | --- |
| RCC | PN-15 | Ventana | Mouse |
| CD3 | 2Gv6 | Ventana | Rabbit |
| CAIX | ab15086 | Abcam | Rabbit |
| CD20 | L26 | Agilent | Mouse |
| CD45 | 2B11+PD7/26 | Agilent | Mouse |
| CD68 | PG-m1 | Agilent | Mouse |
| Ki67 | M7240 | DAKO | Mouse |
| CD31 | M0823 | DAKO | Mouse |
| P53 | M7001 | DAKO | Mouse |
| P-mTOR S2448 | sc293133 | Santa Cruz | Mouse |
| MTCO2 | ab3298 | Abcam | Mouse |

Suppliers: Ventana Medical Systems (Oro Valley, AZ, USA); Abcam (Cambridge, UK); Agilent Technologies (Santa Clara, CA, USA); DAKO GmbH (Jena, Germany); Santa Cruz Biotechnology (Dallas, TX, USA).
